# Prevalence and correlates of stunting among the school-age population in North-Central Nigeria

**DOI:** 10.11604/pamj.2018.31.170.15763

**Published:** 2018-11-09

**Authors:** Idris Abiodun Adedeji, Mohammed Faruk Bashir, David Danjuma Shwe, Collins John

**Affiliations:** 1 Department of Paediatrics, Abubakar Tafawa Balewa University Teaching Hospital, Bauchi, Nigeria; 2 Department of Paediatrics, University of Jos, Nigeria

**Keywords:** Stunting, prevalence, school-age children, correlates, Northern-Nigeria

## Abstract

**Introduction:**

Stunting remains a huge public health concern among developing Nations. However, the burden of this problem among the school-age population appears to have been eclipsed by most nutritional surveys that focus more on the under-fives. This study aimed to demonstrate the prevalence, and identify socio-demographic factors that are associated with stunting among the school-age children in North central Nigeria.

**Methods:**

This was a descriptive cross-sectional study that involved 450 pupils, aged 6-12 years from 10 randomly selected primary schools in Jos, Plateau state. Anthropometric indices were measured using standard techniques and the Height-for-age z-scores were generated using the WHO Anthroplus software. Socio-demographic details were obtained using semi-structured questionnaires. Data were analysed using EPI infoTM statistical software 7.1.5.2.

**Results:**

The mean age of the subjects was 9.3 ± 1.8 years and the male to female ratio was 1:1.1. The prevalence of stunting was 10.5%. The prevalence of stunting was significantly higher among pupils that attended public schools (p<0.0001), those whose mothers had less than secondary level of education (p=0.0427), those between the ages of 10-12 years (p<0.0001), those from the lower socio-economic class (p=0.0021), and those whose family sizes were larger than six family members (p=0.0063).

**Conclusion:**

The substantial burden of stunting among the school age population has significant correlation with certain socio-demographic factors. Addressing these factors by alleviating poverty, promoting maternal literacy and encouraging family planning may, perhaps, lessen the burden of stunting among the school-age group in Northern Nigeria.

## Introduction

Stunting is an evidence of long standing nutritional inadequacy and, it currently constitutes a huge public health concern especially among developing countries [1]. Global estimates show that there are about 162 million stunted children, out of which 32% can be found in Africa [1]. In Nigeria, the prevalence of stunting varies across the different geo-political zones; it ranges from 55% in the North-West to 16% in the South-East [2]. Stunting, which has been said to be the best indicator of overall well-being among children, is associated with poor health, impaired cognitive function, poor school performance and limited economic productivity [3]. Most global and National nutritional surveys focus on children under the age of five years, much to the disadvantage of those in the school-age bracket. The reason for this apparent unevenness may simply be because unlike the school-age group, nutritional status and such other vital data among under-fives are seen as part of National and global core health indicators [4]. Furthermore, there seem to be evidence that providing nutritional intervention in early childhood has more positive effect on developmental outcome than when provided in older children [5]. However, under-nutrition during the school-age period has been shown to significantly impact on both the physical and mental capacity of children [3, 6]. Finding a holistic solution to stunting among the school-age group will entail the identification of the socio-demographic factors that are associated with, and perhaps, perpetuate the condition. Historically, such factors that have been found to be risks for malnutrition among the school-age population include maternal illiteracy and poverty. The aim of our study was to describe the burden of stunting among school-age children between the ages of six and 12 years, as well as to identify the socio-demographic correlates of stunting among these subjects.

## Methods

**Study area:** This study was conducted in Jos, the capital city of Plateau State. Plateau State is located in the North-Central region of Nigeria.

**Study design:** This was a descriptive cross-sectional study that involved 10 primary schools across Jos metropolis.

**Ethical consideration:** Ethical approval was obtained from the Jos University Teaching Hospital research and ethical committee. Approval was obtained from the Local Government Education Authority. While written informed consent was also obtained from the parents/guardians, assent was obtained from the pupils. The study was at no financial cost to the participants.

**Sampling technique:** The minimum sample size was determined using Cochran's sample size formula for categorical data at alpha level of 0.05 and power of 95% (n= z^2^pq/d^2^ ) [7]. Five public and 5 private schools in Jos metropolis were selected randomly. The number of pupils recruited from each school was based on proportionate sampling i.e: Number from each school = (Total population in the school x sample size)/Population in all the 10 selected schools At each school, the pupils were recruited from all the six grades. The number recruited from each grade was also determined by the proportionate sampling method, i.e: Number from each grade = (Total no in the grade x Total no selected from the school)/Total population in the school A list of all the pupils in each grade was subsequently generated and the pupils that took part in the study were eventually selected by using systematic sampling technique. The sampling interval was determined by dividing the total number of pupils in each grade by the number of pupils to be selected from the grade.

**Data collection/study protocol:** Questionnaires were given to the pupils to take home to their parents for completion, and these were returned to the school on the following day. The questionnaires obtained information on social indices, such as family size, parental educational and socio-economic status (using the system suggested by Olusanya *et al.* [8]). Height was measured using a Seca^R^ stadiometer and the reading was taken to the nearest 0.1cm. The weight in kilogram (kg) was measured and read to the nearest 0.1kg.

**Data analysis:** The WHO Anthroplus software (v1.0.4) was used to generate the Height for age z-score (HAZ). This tool is based on the WHO 2007 growth reference. Stunting was defined as HAZ < -2SD. The chi-square test was used to establish the association between categorical variables. All data were processed and analysed using EPI info^TM^ statistical software 7.1.5.2. The level of significance for the chi square test was <0.05. Results are represented on tables.

## Results

**Characteristics of subjects:** Out of the 450 pupils that were enrolled in the study, 410 returned their questionnaires completed. There were 196 males and 214 females, giving a male to female ratio of 1:1.1. Most of the pupils attended private schools (56%) and the mean age of the subjects was 9.3 ± 1.8 years (Table 1).

**Table 1:** Characteristics of subjects

Variable	Male N = 196	Female N = 214	Total N = 410
**School type**			
Public	89	92	181
Private	107	122	229
**Age (years)**			
6	14	16	30
7	23	29	52
8	28	26	54
9	38	32	70
10	39	54	93
11	23	28	51
12	32	29	60
**Mean HAZ**	-0.38±1.29	-0.48±1.35	
**Mean age age SD (years)**	9.3±1.8		

**Correlates of stunting:** Among the subjects, a total of 43 were stunted, giving a prevalence of 10.5%. About 51% of the mothers of the stunted children had no formal education, while 68% of the stunted children had mothers who had only received primary education (Table 2). Most of the stunted pupils (72%) attended public schools while majority (59%) of the non-stunted attended private schools. Seventy-six per cent of the stunted children had parents in the low socio-economic class, while only about 5% of the stunted had parents in the upper socio-economic class. Among the stunted, the older pupils between the ages of 10-12 years were observed to have a higher occurrence (79%) compared to the younger pupils between the ages of six and 9 years (21%) (Table 2 suite). The socio-demographic factors that had significant association with stunting were mothers' educational status (p = 0.0427), school type (p < 0.0001), social class (p = 0.0021), age category of the pupils (p < 0.0001) and family size (p = 0.0063).

**Table 2:** Correlates of stunting

Variable	Stunted N = 43 (%)	Normal N = 367 (%)	Total N = 410 (%)	*Χ^2^*	*P*-Value
**Fathers’ educational status**				2.0673	0.5586
No formal education	15(34.88)	96(26.16)	111(27.07)		
Primary	7(16.28)	52(14.17)	59(14.39)		
Secondary	7(16.28)	81(22.07)	88(21.46)		
Tertiary	14(32.56)	138(37.60)	152(37.07)		
Total	43(100)	367(100)	410(100)		
**Mothers’ educational status**				8.1654	0.0427
No formal education	22(51.16)	128(34.88)	150(36.59)		
Primary	7(16.28)	38(10.35)	45(10.98)		
Secondary	8(18.60)	90(24.52)	98(23.90)		
Tertiary	6(13.95)	111(30.25)	117(28.54)		
**Total**	43(100)	367(100)	410(100)		
**Fathers’ age (years)**				1.9234	0.3822
20-39	13(30.23)	129(35.15)	142(34.63)		
40-60	22(51.16)	196(53.41)	218(53.17)		
>60	8(18.60)	42(11.44)	50(12.20)		
**Total**	43(100)	367(100)	410(100)		
**Mothers’ age (years)**				0.3348	0.8458
20-39	29(67.44)	255(69.48)	284(69.27)		
40-60	11(25.58)	81(22.07)	92(22.44)		
>60	3(6.70)	31(7.56)	34(8.29)		
**Total**	43(100)	367(100)	410(100)		

**Table 2 (suite):** Correlates of stunting

Variable	Stunted N=43 (%)	Normal N=367 (%)	Total N=410 (%)	*Χ^2^*	P-Value
**School type**				15.216	<.0001
Public	31(72.09)	150(40.87)	181(44.15)		
Private	12(27.91)	217(59.12)	229(55.85)		
**Total**	43(100)	367(100)	410(100)		
**Age category (years)**				16.5119	<.0001
6-9	9(20.93)	197(53.68)	206(50.24)		
10-12	34(79.07)	170(46.32)	204(49.76)		
Total	43(100)	367(100)	410(100)		
**Social-class**				12.337	0.0021
Upper	2(4.65)	66(17.98)	68(16.59)		
Middle	8(18.60)	121(32.97)	129(31.46)		
Lower	33(76.74)	180(49.04)	213(51.95)		
**Total**	43(100)	367(100)	410(100)		
**Family size**				7.4419	0.0063
≤6	19(44.19)	240(65.40)	259(63.17)		
>6	24(55.81)	127(34.60)	151(36.83)		
Total	43(100)	367(100)	410(100)		
**Family type**				1.839	0.1751
Monogamous	25(58.14)	251(68.39)	276(67.32)		
Polygamous	18(41.86)	116(31.60)	134(32.68)		
**Total**	43(100)	367(100)	410(100)		

## Discussion

The prevalence of 10.5% recorded for stunting in this study is similar to the findings from earlier studies among the school age population [9, 10]. Abah *et al.* and Akor *et al.* who similar to our work, carried out their studies in North-Central Nigeria, recorded a stunting prevalence of 10.3% and 11.4% respectively [9, 10]. However, in another study done in Makurdi, North-Central Nigeria by Goon *et al* the prevalence of stunting among the school age children was found to be 52% [11]. The explanation for this wide disparity may be because Goon et al studied only children between the ages of 9-12 years. Thus, the higher mean age of the subjects in their study may have contributed to their finding, as it has been shown that older school age pupils, especially the early adolescents, are more likely to suffer from under nutrition [12]. In Enugu, South East Nigeria, significantly lower prevalence of 0.8% and 0.4% were documented by Igbokwe *et al* and Eze *et al.* [13, 14]. This lower values may be explained by the fact that situations that have been consistently shown to be associated with a higher burden of childhood malnutrition are known to be more prevalent in the Northern part of Nigeria, than in the South Eastern region [2]. Such include illiteracy and poverty [2]. Outside Nigeria, Dabone *et al* in Ougadogou, Burkina Faso reported a prevalence of 8.3%, while Appiah and Amos in Volta region, Ghana documented a higher prevalence of 50% for stunting among the school-age population [15, 16]. The much higher prevalence of stunting in the latter work may also be as a result of the higher mean age of the pupils (13 years) in their study.

Our study demonstrated a significant relationship between the educational status of the mother and the occurrence of stunting. We realised that among the stunted, about two-thirds had mothers who had received less than secondary level of education and about half had mothers who had no formal education. Maternal literacy has a strong influence on a child's nutritional status, as revealed by many earlier studies [17-20]. A literate mother is more likely to have a better understanding of basic nutrition and healthcare and is also more likely to be assertive in making sound health related decisions. Furthermore, a literate mother is more likely to be economically empowered and independent. Ultimately, such mothers are better positioned to take care of their children. According to the 2013 National Demographic Health survey (NDHS), the likelihood of malnutrition is more than three times higher in children whose mothers had not received a formal education [2]. A similar trend was observed in the earlier reports by Sufiyan *et al.* in Zaria and Ighogboja in Jos, who both observed that about 65% of the mothers of stunted children had no formal education [17, 18]. Senbanjo *et al.* and Owoaje *et al.* also made similar observations in their studies in South West Nigeria, as they concluded that low parental education was a significant risk factor of childhood malnutrition [19, 20]. The early stages of adolescence are characterised by rapid growth and development, with the attendant increase in metabolic activity and energy demand [21]. Thus, in situations whereby there is nutritional inadequacy, school age children at this stage are at a higher risk of becoming malnourished. This assertion was buttressed by our study, as we recorded a significantly higher prevalence of stunting among children between the ages of 10-12 years. Fetuga *et al.*, as well as Abah *et al.* made similar observations [9, 12].

This study found a significant association between school type and stunting. Seventy-two per cent of the stunted children were found in non-fee paying government owned schools. Indeed, unlike in the private schools where we recorded a prevalence of 5%, the prevalence of stunting among the pupils in public schools was 17%. This is similar to the earlier observations of Agbo *et al.* and Akor *et al.*, who had also recorded a greater percentage of malnourished pupils in public schools [10, 22]. Most children in non-fee paying schools are more likely to have parents who are in the low socio-economic class. Such parents are less likely to be able to afford the food items required for optimal nutrition. Furthermore, similar to other studies, we found that socio-economic class had a significant relationship with stunting [2, 13, 18-20]. In fact, 76% of the pupils that were stunted had parents in the lower socio-economic class. However, the higher prevalence of stunting among the children of parents in the low social class may not be ascribable wholly to their limited purchasing power of nutritious food items; it may also be possible that such parents are ignorant of the importance of adequate dietary requirement among children in the school age group. This is even more so, since the literacy level of the parents also determines their socio-economic status [8]. A family size of greater than four children was also found to be significantly associated with a greater prevalence of stunting. This is perhaps a challenge in families where resources are limited and with no food security. The study by Owoaje *et al.*, also found that children from families with greater than four children had 4-folds greater risk of becoming malnourished [20].

## Conclusion

This study has demonstrated that there is a significant burden of stunting among the school-age children. Socio-demographic indices such as maternal illiteracy, poverty, lack of family planning are factors that may continue to potentiate stunting among the school-age. More attention needs to be paid to pupils in the early phase of adolescence, as this group has been observed to be exceptionally at risk of becoming malnourished. Ultimately, curbing stunting among the school age is of paramount priority, as this will allow children attain their maximum potentials. The approach to solving the problem is multidisciplinary and revolves around poverty alleviation and improving maternal literacy.

### What is known about this topic

- It is already an established fact that there is a significant burden of chronic childhood under-nutrition, in Northern Nigeria.

### What this study adds

- This study has shown that beyond the under-fives, who are the targets of most nutritional surveys in Northern Nigeria, the school age children in this region of the country also have a significant burden of stunting;
- Furthermore, the study also revealed that those previously established risk factors for under-five malnutrition equally have significant association with the occurrence of stunting among the school-age population.

## Competing interests

The authors declare no competing interests.
